# Landauer’s formula with finite-time relaxation: Kramers’ crossover in electronic transport

**DOI:** 10.1038/srep24514

**Published:** 2016-04-20

**Authors:** Daniel Gruss, Kirill A. Velizhanin, Michael Zwolak

**Affiliations:** 1 Center for Nanoscale Science and Technology, National Institute of Standards and Technology, Gaithersburg, MD 20899, USA; 2 Maryland Nanocenter, University of Maryland, College Park, MD 20742, USA; 3 Department of Physics, Oregon State University, Corvallis, OR 97331, USA; 4 Theoretical Division, Los Alamos National Laboratory, Los Alamos, NM 87545, USA.

## Abstract

Landauer’s formula is the standard theoretical tool to examine ballistic transport in nano- and meso-scale junctions, but it necessitates that any variation of the junction with time must be slow compared to characteristic times of the system, e.g., the relaxation time of local excitations. Transport through structurally dynamic junctions is, however, increasingly of interest for sensing, harnessing fluctuations, and real-time control. Here, we calculate the steady-state current when relaxation of electrons in the reservoirs is present and demonstrate that it gives rise to three regimes of behavior: weak relaxation gives a contact-limited current; strong relaxation localizes electrons, distorting their natural dynamics and reducing the current; and in an intermediate regime the Landauer view of the system only is recovered. We also demonstrate that a simple equation of motion emerges, which is suitable for efficiently simulating time-dependent transport.

The prototypical example of electron transport in nano- and meso-scale junctions is a small conducting region connected to two electron reservoirs. When the confinement in this region is strong, the rigorous treatment of quantum effects becomes crucial. The Landauer formalism^1^,^2^ is a well-known method for describing these systems, which is based on an energy-dependent transmission probability for the region of interest. This method has been successfully applied to ballistic transport^3^, quantized conductance^4^,^5^, quantum point contacts^6^, cold-atom systems^7^,^8^, and broadly in the area of nanoscale electronics^2^,^9^,^10^. However, the viewpoint on which the Landauer formula is based neglects the explicit effect of relaxation mechanisms, the dynamics of the region of interest, and many-body interactions. Put differently, the text-book Landauer approach^2^,^11^,^12^ implicitly assumes that deviation from the equilibrium distribution in the external reservoirs is negligible. There is thus an interplay between relaxation timescales - e.g., those in the junction and at the interface with the electrodes - that cannot be captured by the Landauer formalism.

An alternative method to calculate the transport properties is to work with a closed system and explicitly solve for the dynamics^2^,^13^,^14^,^15^. This approach has been applied to study molecular conductance^16^,^17^ and induced cold atom transport^8^,^18^,^19^, where the latter closely approximates a closed system, giving an ideal application of this approach. The limitation of this method, however, is that the recurrence time is proportional to the total system size, meaning that a large—and computationally expensive—reservoir is needed to fully eliminate transient effects and to examine dynamical perturbations on top of an otherwise steady-state current. This is not feasible in most situations, especially if one is interested in complex, time-dependent many-body systems or the effect of relaxation (which would require the explicit incorporation of additional degrees of freedom such as phonons).

Transport in time-dependent structures, however, has emerged at the forefront of applications. Electronic sequencing^20^,^21^,^22^,^23^,^24^,^25^,^26^,^27^ (via tunneling current through base pairs) and sensing^28^,^29^,^30^,^31^,^32^,^33^,^34^,^35^ (e.g., protein fluctuations on/nearby carbon nanotube and graphene devices), in particular, require a rigorous treatment of the interplay between transport and changes of the junction or molecular structure. In other words, if the local relaxation of electrons near the junction is slower or on the same timescale as the changes in the junction (typically picoseconds) the interaction between these two processes can dramatically influence transport. Practical real-time approaches to transport that are naturally suited to these systems are therefore necessary. The formalism introduced by Jauho, Meir and Wingreen^36^,^37^,^38^ (which has been used to describe the conduction in a variety of systems, such as quantum dots^39^,^40^,^41^, and layered semiconductors^42^,^43^,^44^) provides an exact formal solution to the time evolution, but involves two-time Green’s functions, making its use prohibitive in many applications. In this report, we show that in the absence of time dependence, one can recover the Landauer view with reasonably sized “extended reservoirs” and weak relaxation. The incorporation of explicit but finite reservoirs, however, also allows for one to examine the competition between time-dependence of the junction and the relaxation rate of the reservoir region.

In transport, external sources and sinks of electrons, together with electron-electron, electron-phonon, etc., interactions, seek to sustain an equilibrium potential difference across two large regions, which we call extended reservoirs. We develop an open system approach to transport that includes a finite electron lifetime representing the presence of these relaxation mechanisms. In particular, the extended reservoirs consist of a set of states whose occupation is pushed towards equilibrium by the exchange of electrons with *implicit reservoirs* (the environment 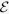) at different chemical potentials. When a finite system is placed between them, an electric current will be driven across it. We derive a Landauer-like formula for this scenario and demonstrate that a finite relaxation time in the extended reservoirs gives rise to three distinct regimes of behavior, analogous to Kramers’ turnover for chemical reactions^45^. A methodology similar to that below—one based on the concept of “extended reservoirs”—was proposed and developed for classical thermal transport^46^,^47^, where a corresponding crossover effect occurs (see also ref. 48).

## Results

### Model

**Figure 1:**
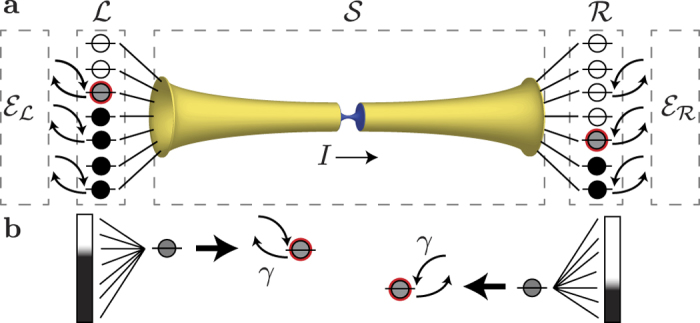
Schematic representation of the model. (**a**) System-reservoir-environment model, with yellow and blue representing the junction region (e.g., two leads connected by a junction)—the system of interest 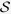
—and 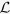 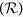 indicating the extended reservoirs. The presence of electron sources, sinks, and interactions (electron-electron, electron-phonon, etc.), here subsumed into the environments 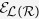, causes the reservoirs to relax toward their respective equilibrium distributions, which, when an external bias is applied, will be at different chemical potentials. (**b**) Each reservoir state exchanges electrons with an environment (i.e., an external reservoir at some chemical potential), which gives rise to a non-zero relaxation rate *γ*. The imbalance of occupied states will drive a current through 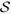, where explicit (or implicit) relaxation mechanisms may or may not be present.

The Hamiltonian is 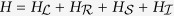, where the explicit degrees of freedom are divided into three parts: the left extended reservoir (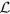), the right extended reservoir (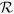), and the system of interest (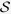). The extended reservoir regions have a finite electron lifetime that pushes them towards equilibrium by allowing for the exchange of electrons with the external degrees of freedom in the implicit reservoir. In some sense, we can say this is a grand canonical approach to transport when compared to the microcanonical approach of ref. 13. In other words, 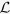 and 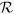 are open to some larger environment 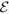 (shown as 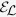 and 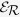 in Fig. 1), where the latter will be composed of degrees of freedom that are treated implicitly. Finally, 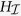 describes the interaction between 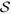 and the left (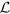) and right (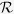) extended reservoirs. Figure 1 shows a schematic of this setup.

The left and right regions each contain *N*_*r*_ non-interacting electronic states with a Hamiltonian given by 
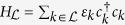
 and 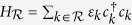, where 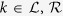 indexes the single particle states and 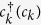 are their respective creation (annihilation) operators. The interaction Hamiltonian is described by 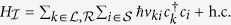, where 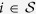 indexes the system states (with associated operators 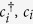). The *v*_*ki*_ are the hopping rates between the reservoir and system states. The method we describe will be applicable to all dimensions, as this just changes the onsite energies in the Hamiltonian and hopping rates to the extended reservoirs. The system Hamiltonian, 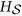, is arbitrary, potentially including many-body or spin-dependent interactions, vibrational degrees of freedom, etc.

In the absence of 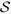, the extended reservoir states relax into their equilibrium occupations, i.e., their local density of electrons decays into a Fermi-Dirac distribution. The rate at which this occurs, denoted by *γ*, is controlled by the coupling strength between the reservoirs, 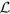 and 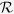, and their environment, 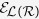. Generically, *γ* captures the physical interaction with the environment that relax the reservoirs into equilibrium. The relaxation rate can also be energy dependent and this will reflect both the geometry and dimensionality of the whole setup. In this work, we take *γ* to be constant, independent of both *k* and 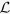 or 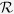, for simplicity. This is easily relaxed, however. A lower bound on *γ*^−1^ can be estimated by the mean scattering time in the material, which is typically on the order of 1 fs to 10 fs for metals. However, *γ* is the relaxation rate to reach equilibrium, which can be much weaker (especially for, e.g., local disturbances to dissipate in confined geometries, at low temperature, or in the presence of weak electron-phonon interaction). Physically, each reservoir state is exchanging electrons with a larger external reservoir (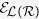) with an applied bias of 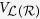 and an infinite extent. The total externally applied bias is 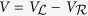, where we here take *V* to be in units of energy.

The general solution for the steady-states in this setup can be found by following the approach of Jauho, Meir, and Wingreen^37^, where the reservoirs are taken to be infinite with a well-defined occupation and no relaxation. Indeed, when the whole 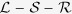 system is treated as some larger system 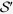, the steady state is just the Meir-Wingreen solution, albeit with an unmanageably large number of degrees of freedom. Here, however, we are interested in calculating the transport properties of 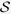 *by itself*, i.e., to what extent can the extended reservoirs 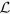 and 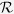
—finite in extent but with relaxation—capture the effect of infinite reservoirs in the normal approaches. As well, we want to determine what parameter ranges (e.g., realistic values of *γ*) are simulatable via a Markovian master equation approach. To this end, we will start with the Green’s functions for the extended reservoir states uncoupled from the system, but still including a finite lifetime (note that, as we do in the Supplemental Information, one can start with all degrees of freedom treated explicitly, including 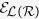, see Eq. (A1)). For the lesser Green’s function:


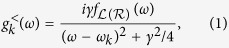


with 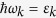 and 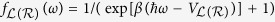 is the Fermi-Dirac distribution. This expression is within the wide-band approximation, see the Supplemental Information for the general case. This leads to the single particle retarded and advanced Green’s functions


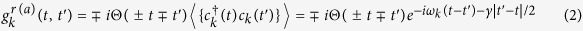


or 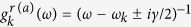 for the Fourier transform. The *γ* in both these equations reflects the finite lifetime of electrons in the extended reservoir regions. Starting with this broadened Green’s function for the individual reservoir state, the steady-state current is


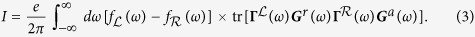


The quantity 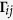 is the spectral density of the couplings between the system and the extended reservoirs


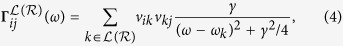


with 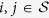. 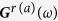 are the exact retarded and advanced Green’s functions for the system only but in the presence of the left and right extended reservoirs and including relaxation in the latter.

Equation (3) is similar to the traditional Landauer formula. However, the density of states of a single extended reservoir state is broadened to a Lorentzian due to the inclusion of a finite relaxation time. When no interactions are present, Eq. (3) can be interpreted in terms of a relaxation-dependent transmission coefficient, 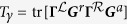. This relaxation gives rise to different physical regimes of behavior and also an effective equation of motion that can be used to examine transport in more complex time-dependent scenarios. We first describe the different regimes of behavior for an example system.

When the reservoir states are symmetrically coupled to the system, i.e., when the distribution of energies *ε*_*k*_ and couplings *v*_*ki*_ are the same for each 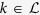 and its corresponding 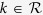, the spectral density, Eq. (4), is proportional to the imaginary part of the inverse of ***G***^*r*(*a*)^(*ω*). This results in a simplified expression for the current,


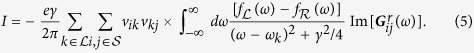


In the example below, we make use of this simplified expression.

### Single-Site Homogeneous System

Equation (3) is valid for any system—including those with many-body interactions—with a finite relaxation time. In what follows, however, we will focus on a homogeneous system in which the combined 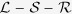 system is a 1D lattice with hopping rate *J*: 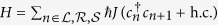. The quantity *J* sets the frequency scale, where the bandwidth *W* = 4*J*. Note that in this example, the total coupling to the system and the bandwidth are both determined by *J*. Typically, *J*^−1^ is in the range 0.1 fs to 1 fs for conducting materials. We choose to work with hopping rates rather than energies as this gives more transparent expressions.

The extended reservoir portion of *H* can be directly diagonalized via a sine transformation. The transformation 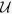 is applied to the subset of states in the reservoirs, and so the couplings to 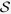 are determined by its matrix elements. That is, given 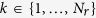, 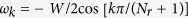. We can express the couplings with a single index, *v*_*k*_ for 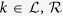 (instead of *v*_*ki*_). Using this notation, the couplings are


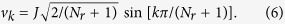


Again, in this special case of a uniform 1D lattice, *J* sets the hopping rate in both the extended reservoir region and between the system and extended reservoirs. Additionally, we will take the system to be a single site with no onsite energy so that 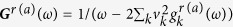, where the sum over *k* is in either 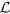 or 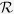 (the factor of 2 reflects the symmetry of the setup).

**Figure 2:**
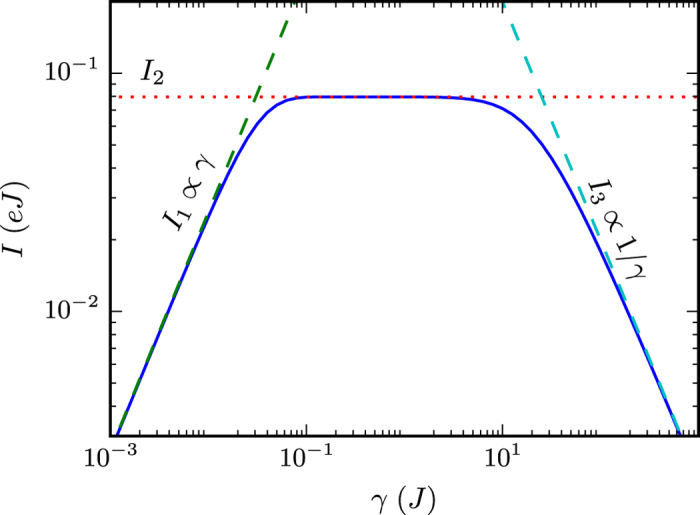
Regimes of the electronic current. The steady-state current, Eq. (5) (or Eq. (3)), of the single-state system connected to two 1D extended reservoirs of size *N*_*r*_ = 64. The potential difference is *V* = 0.5*Jħ* and the temperature is given by *β* = 40(*Jħ*)^−1^. The dashed lines show the approximations in the small and large *γ* regimes, and the dotted line is the Landauer calculation of the closed system, 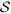, with infinite 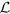 and 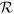 without relaxation. The small *γ* regime has a current increasing linearly with *γ*, as it dominates the rate at which electrons flow through the whole setup. In the large *γ* regime, the fast relaxation localizes electrons in the extended reservoir, causing the current to decay as 1/*γ*. In the intermediate relaxation regime, the current matches that from a Landauer calculation.

Figure 2 shows the calculation of the current *I* from Eq. (5) (or Eq. (3)) as a function of the relaxation rate *γ* for a reservoir size *N*_*r*_ = 64. There are three regimes visible: (1) a small *γ* regime with current *I*_1_, (2) an intermediate regime with *I*_2_, and (3) a large *γ* regime with *I*_3_. We first discuss the intermediate regime.

### Intermediate *γ*

**Figure 3:**
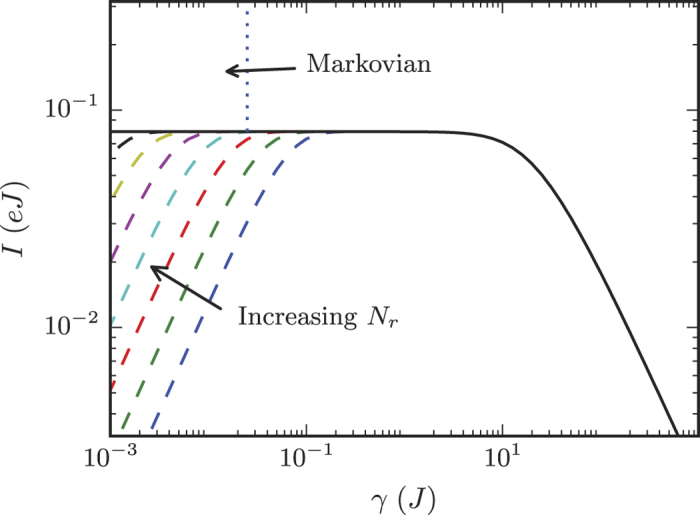
Expansion of the plateau. The steady-state current as a function of the relaxation rate *γ* for the 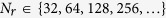 systems (dashed lines) and the *N*_*r*_ → ∞ limit, *I*_23_ (solid line). The parameters of the system are the same as in Fig. 2. In the limit that *N*_*r*_ → ∞ and then *γ* → 0, we recover the standard Landauer current. The vertical dotted line demarcates the regions where the Markovian master equation is valid and not valid. Since the size of the plateau grows linearly with *N*_*r*_, the plateau will eventually extend into the region where the Markovian equation is valid, allowing for transport in this intermediate plateau regime to be simulated with the much simpler Markovian approach.

Figure 2 shows that there is an intermediate range of *γ* for which the current is approximately flat. That is, in this crossover region between small and large values of *γ*, a plateau forms and subsequently elongates as the size of the extended reservoir increases (see Fig. 3). The current in this regime is the same as that predicted by a Landauer calculation for 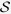 alone. That calculation gives the current as 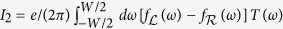. In linear response, this yields


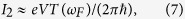


where *ħω*_*F*_ is the Fermi level. In this example, the transmission coefficient at the Fermi level is *T*(*ω*_*F*_) = 1 and the plateau comes at the quantum of conductance, 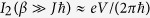 (the transmission coefficient through part of a homogeneous lattice is unity, *T*(*ω*) = 1, for all frequencies in the band). At high temperature the current is 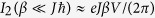.

Perfect transmission at the Fermi level (*T*(*ω*_*F*_) = 1) remains even if the hopping rate from the extended reservoir into the system is different (i.e., even if we have an inhomogeneity of the hopping rates at the interface to the system). In other systems, or in nonlinear response, though, the current—i.e., the level of the plateau—will be a complicated function of the total setup. As we discuss below, this will change when the current transitions into the other two regimes.

### Small *γ*

Figure 2 shows that the current increases linearly with *γ* when it is small. In this regime, electrons move from the left extended reservoir into the system much faster than the implicit reservoirs replenish the electrons in 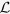 (and similarly for 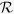). The rate of the replenishment is the relaxation rate *γ*, as this determines how fast the states return back to their equilibrium occupation. Thus, when *γ* is small, electrons cannot be restored rapidly enough and this rate becomes the bottleneck for the current, and hence the current is essentially dependent only on *γ*.

When the system is noninteracting and *γ* is much less than the state spacing (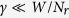), energy conservation guarantees that an electron coming out of state *k* (i.e., at energy *ε*_*k*_) on the left, must exit the system at the same energy on the right. This allows the current to be broken into contributions from pairs of states, for which the pair can also be labeled by *k* in this symmetric setup. The current flowing into the left reservoir state *k* from the environment 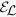 is 
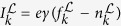
 and the current out of that state into the system is 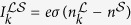, where 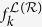 is the Fermi-Dirac distribution evaluated at the reservoir state frequency 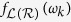 and *σ* is the particle flow rate from the reservoir state into the system. Similar rate equations hold on the right side. In the steady state (
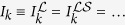
) and when *γ* ≪ *σ* (i.e., the relaxation *γ* has to be weak enough that electrons are injected into an extended reservoir state much more slowly than they move into the system and are subsequently taken away from the interface between the system and extended reservoir), these equations give 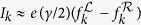, where *γ*/2 is the “reduced *γ*” (i.e., it reflects that there are two interfaces, one at the left and one at the right. For different relaxation rates in 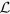 and 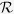, the relevant quantity would be 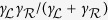). Summing over the contribution from all states *k*, the total current in this regime is


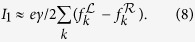


The sum over *k* is over a single set of states in the left or right, which are identical in the symmetric setup. Figure 2 plots Eq. (8) along with the full solution, showing agreement for small *γ*.

Essentially, Eq. (8) is just *eγ*/2 times a particle bias: There are 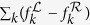 open channels in the bias window—where an electron can move from an occupied state *k* on the left and go to an unoccupied state *k* on the right—and each contributes *eγ*/2 to the current. We note that the physics of this regime is the same as that observed in weakly coupled quantum dot systems^49^, in which case *γ* reflects a weak tunneling rate to the external electrodes which limits how fast the dot at the boundary can equilibrate with the electrode.

The transition from the small to intermediate regimes occurs when the current from Eq. (8) intersects the plateau current, Eq. (7). We can approximate Eq. (8) by *eγ*/2(*V*/*ħ*)*N*_*r*_/*W*, where *V*/*ħ* is the bias window in terms of frequency, *W*/*N*_*r*_ is the frequency spacing of the reservoir states, and, thus 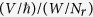 gives the number of states in the bias window. Note that *N*_*r*_ should also be sufficiently large so that a significant number of states are within the bias window. The *γ* at which the transition occurs, which we will denote by *γ*_12_, is


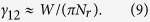


This value decreases inversely with *N*_*r *_. Indeed, as seen in Fig. 3, this is responsible for the increasing size of the plateau region, as the transition to the large *γ* region is independent of *N*_*r*_ (which we will see below).

We note that this transition *γ* is equivalent to the condition necessary to be in the small gamma regime, 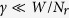. In more complex systems, or even just in nonlinear response, the transition *γ* can be dependent on many other factors besides just the mode spacing, such as the hopping rate to the system, the bias, etc. In other words, the transition from small to intermediate *γ* depends on the details of the setup.

### Large *γ*

When *γ* becomes large, Fig. 2 shows that the current “turns over” and starts to decay as 1/*γ*. The strong relaxation (i.e., the fast relaxation rate) in this regime is effectively localizing electrons in the extended reservoir region. For currents to flow, electrons must remain coherent between the extended reservoir and the system. The relaxation limits this coherence to a time ≈1/*γ* and therefore the current is suppressed by this factor.

Alternatively, this can be seen by starting with Eq. (5). There, the Lorentzian is approximately constant (1/*γ*) in the relevant region of integration and the Green’s functions for the reservoir states, 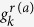, are purely imaginary. The density of states, 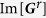, is dominated by the contribution from the system in this example (see the Supplemental Information for more details). In linear response, this gives


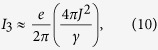


so long as *V* ≠ 0. That is, the strong relaxation renormalizes the coupling to *J*^ 2^/*γ* and, thus, the total electron flow through 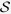 is limited by this factor. This expression depends on the hopping rate to the system rather than the hopping rate in the extended reservoirs. As we show in the Supplemental Information, the current is related to the difference in real space occupation of the sites immediately adjacent to 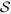 for the Markovian approach discussed below. This also shows that the current in the large *γ* regime is independent of *N*_*r*_, with the exception of potential discretization effects (when *N*_*r*_ is very small) that can cause mismatches in energy, and that the current is independent of the bias in this regime for the particular example we discuss.

Just as with the small *γ* regime, we can find the transition into the large *γ* regime. This occurs at 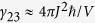, where we have denoted the transition *γ* as *γ*_23_. Thus, while the behavior of the current in the large *γ* regime is independent of bias, the transition to this regime is dependent on the bias—decreasing the bias makes this transition occur at increasingly large values of *γ*.

We note that, unlike *γ*_12_ and *γ*_23_, how the small and large *γ* behavior varies with *γ* is generally independent of the form of the system and the reservoir dispersion relation, but rather only depends on characteristic quantities such as the total coupling strength (between the system and extended reservoir) and relaxation rate.

### *N*_*r*_ → ∞ Limit

The *N*_*r*_ → ∞ limit can be taken in Eq. (3) to regain a macroscopic electron reservoir, but with a finite relaxation time. In our example setup, the extended reservoirs become semi-infinite 1D lattices on each side. For this case, we can find the self-energy through either a recursion relation^8^,^50^ or by integrating the states directly:


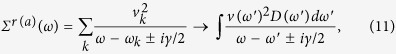


with 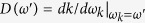. This expression gives a self-energy


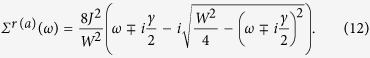


Using this in Eq. (3) or Eq. (5) (with **Γ** = 2Im∑) provides a semi-analytic expression for the exact current through the system in the infinite *N*_*r*_ limit, denoted by *I*_23_. Figure 3 shows this quantity together with the solution for several finite *N*_*r*_ reservoirs. In addition, the *N*_*r*_ → ∞ result can be expanded for small *γ*, yielding the lowest order contribution to the current as given by Landauer, 
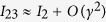
. These show that as *N*_*r*_ increases the plateau will continually grow and, when *N*_*r*_ → ∞, the small *γ* regime will be eliminated entirely.

The results above are for steady-state currents, which can be calculated from exact treatment of the 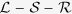 system. However, this neglects time-dependent effects present in 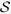. As we show in the Supplemental Information, Eq. (3) also describes the steady-state solution of the Markovian master equation


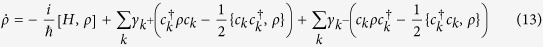


in the small *γ* regime and in part of the intermediate plateau region (so long as *N*_*r*_ is sufficiently large, see Fig. 3). This type of equation has been applied previously^51^,^52^,^53^,^54^. It is often taken as a phenomenological equation for all regimes of *γ*, not as a weak-coupling approximation to a memory-less reservoir^55^. Our complete solution to both the full model (for all *γ*) and its Markovian counterpart enables us to put rigorous bounds on the latter’s validity, which we will now discuss.

In Eq. (13), the terms 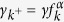 and 
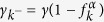
, where 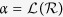 when 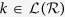, relax the extended reservoirs into an equilibrium defined by their isolated Hamiltonian when 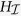 is absent. That is, unlike the setup described above, this equilibrium is for the extended reservoir states at fixed energy 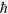
ω_*k*_. This coincides with the concept of equilibrium above only when the broadening is sufficiently small. Larger *γ*, therefore, can give rise to unphysical behavior, such as residual currents at zero bias (see the Supplemental Information). In particular, the relaxation in the extended reservoirs must be smaller than the thermal relaxation, 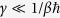 (or, in terms of timescales, *γ*^−1^ ≫ 25 fs at room temperature), otherwise electron occupation can be smeared well above the Fermi level. As well, if one has asymmetric 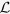 and 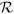 extended reservoirs—with the asymmetry characterized by an energy offset *δ* (see the Supplemental Information)—one needs 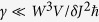. Taking *γ* and *N*_*r*_ such that the current is on the plateau, *γ* ≈ *W*/*N*_*r*_, gives a requirement on the extended reservoir size, 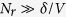, when *δ* is finite. Without an asymmetry, the anomalous current from the left reservoir to the right is canceled by the anomalous current from right to left. This less strict condition (when compared to 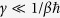) guarantees that superfluous currents will be negligible compared to the actual current at finite bias. Within these regimes, the Markovian master equation allows for the calculation of the *full time dynamics*. This formalism allows for the simulation of time-dependent effects or interactions and, notably, does so without the use of two-time Green’s functions or the use of memory kernels, which both drastically increase the complexity of the simulations.

## Discussion

In summary, we developed the concept of extended reservoirs to examine the effect of relaxation on transport and the validity of a Markovian master equation approach. In addition to providing the full, exact solution to both the Markovian and non-Markovian cases, we showed that the current displays a crossover behavior as the relaxation rate is varied, with a weak coupling limit proportional to *γ* and a strong coupling limit proportional to 1/*γ*. These two regimes are “relaxation” dominated. The Landauer regime can be simulated through the use of a finite number of reservoir states and controlling the relaxation rate to be between these two regimes. The physical behavior in the presence of a finite reservoir is analogous to Kramers’ problem and thermal transport^46^,^47^.

This approach naturally leads to the Markovian master equation, Eq. (13), for small-to-intermediate *γ*, which gives a suitable starting point for studying the real-time behavior of the current where the junction region is time-dependent. This formalism allows the electronic reservoirs to respond to dynamical components of the system (such as structural and energetic fluctuations) and relax back to equilibrium at a finite rate. The method, therefore, can be applied to help understand the role of fluctuations in determining transport properties, to assess the effectiveness of electronic sensing in aqueous solution, and to give a unified approach to simulating nanoscale devices out of equilibrium.

## Additional Information

**How to cite this article**: Gruss, D. *et al*. Landauer’s formula with finite-time relaxation: Kramers’ crossover in electronic transport. *Sci. Rep.* **6**, 24514; doi: 10.1038/srep24514 (2016).

## Supplementary Material

Supplementary Information

## References

[b1] Landauer R. Spatial variation of currents and fields due to localized scatterers in metallic conduction. IBM Journal of Research and Development 1, 223–231 (1957).

[b2] Di Ventra M. Electrical transport in nanoscale systems (Cambridge University Press, Cambridge, 2008).

[b3] Szafer A. & Stone A. D. Theory of quantum conduction through a constriction. Phys. Rev. Lett. 62, 300 (1989). 10040197 10.1103/PhysRevLett.62.300

[b4] Van Wees B. . Quantized conductance of point contacts in a two-dimensional electron gas. Phys. Rev. Lett. 60, 848 (1988). 10038668 10.1103/PhysRevLett.60.848

[b5] Ohnishi H., Kondo Y. & Takayanagi K. Quantized conductance through individual rows of suspended gold atoms. Nature 395, 780–783 (1998).

[b6] Ando T. Quantum point contacts in magnetic fields. Phys. Rev. B 44, 8017 (1991). 10.1103/physrevb.44.8017 9998733

[b7] Brantut J.-P. . A thermoelectric heat engine with ultracold atoms. Science 342, 713–715 (2013). 24158905 10.1126/science.1242308

[b8] Chien C.-C., Di Ventra M. & Zwolak M. Landauer, Kubo, and microcanonical approaches to quantum transport and noise: A comparison and implications for cold-atom dynamics. Phys. Rev. A 90, 023624 (2014).

[b9] Das Sarma S., Adam S., Hwang E. H. & Rossi E. Electronic transport in two-dimensional graphene. Rev. Mod. Phys. 83, 407–470 (2011).

[b10] Aradhya S. V. & Venkataraman L. Single-molecule junctions beyond electronic transport. Nature Nanotech. 8, 399–410 (2013). 10.1038/nnano.2013.91 23736215

[b11] Datta S. Electronic transport in mesoscopic systems (Cambridge university press, 1997).

[b12] Scheer E. Molecular electronics: an introduction to theory and experiment (World Scientific, 2010).

[b13] Di Ventra M. & Todorov T. N. Transport in nanoscale systems: the microcanonical versus grand-canonical picture. J. Phys. Condens. Matter 16, 8025 (2004).

[b14] Stefanucci G. & Almbladh C.-O. Time-dependent quantum transport: An exact formulation based on TDDFT. Europhys. Lett. 67, 14 (2004).

[b15] Bushong N., Sai N. & Di Ventra M. Approach to steady-state transport in nanoscale conductors. Nano lett. 5, 2569–2572 (2005). 16351217 10.1021/nl0520157

[b16] Cheng C.-L., Evans J. S. & Van Voorhis T. Simulating molecular conductance using real-time density functional theory. Phys. Rev. B 74, 155112 (2006).

[b17] Evans J. S., Cheng C.-L. & Van Voorhis T. Spin-charge separation in molecular wire conductance simulations. Phys. Rev. B 78, 165108 (2008).

[b18] Chien C.-C., Zwolak M. & Di Ventra M. Bosonic and fermionic transport phenomena of ultracold atoms in one-dimensional optical lattices. Phys. Rev. A 85, 041601 (2012).

[b19] Chien C.-C., Gruss D., Di Ventra M. & Zwolak M. Interaction-induced conducting-non-conducting transition of ultra-cold atoms in one-dimensional optical lattices. New J. Phys. 15, 063026 (2013).

[b20] Zwolak M. & Di Ventra M. Electronic signature of DNA nucleotides via transverse transport. Nano Lett. 5, 421–424 (2005). 15755087 10.1021/nl048289w

[b21] Lagerqvist J., Zwolak M. & Di Ventra M. Fast DNA sequencing via transverse electronic transport. Nano Lett. 6, 779–782 (2006). 16608283 10.1021/nl0601076 PMC2556950

[b22] Zwolak M. & Di Ventra M. Colloquium: Physical approaches to DNA sequencing and detection. Rev. Mod. Phys. 80, 141 (2008).

[b23] Branton D. . The potential and challenges of nanopore sequencing. Nat. Biotechnol. 26, 1146–1153 (2008). 18846088 10.1038/nbt.1495 PMC2683588

[b24] Chang S. . Electronic signatures of all four DNA nucleosides in a tunneling gap. Nano Lett. 10, 1070–1075 (2010). 20141183 10.1021/nl1001185 PMC2836180

[b25] Tsutsui M., Taniguchi M., Yokota K. & Kawai T. Identifying single nucleotides by tunnelling current. Nature Nanotech. 5, 286–290 (2010). 10.1038/nnano.2010.42 20305643

[b26] Huang S. . Identifying single bases in a DNA oligomer with electron tunnelling. Nature Nanotech. 5, 868–873 (2010). 10.1038/nnano.2010.213 PMC4121130 21076404

[b27] Ohshiro T. . Single-molecule electrical random resequencing of DNA and RNA. Sci. Rep. 2, 501 (2012). 22787559 10.1038/srep00501 PMC3392642

[b28] Willard D. M., Mutschler T., Yu M., Jung J. & Van Orden A. Directing energy flow through quantum dots: towards nanoscale sensing. Anal. Bioanal. Chem. 384, 564–571 (2006). 16440194 10.1007/s00216-005-0250-z

[b29] Choi Y. . Single-molecule lysozyme dynamics monitored by an electronic circuit. Science 335, 319–324 (2012). 22267809 10.1126/science.1214824 PMC3914775

[b30] Goldsmith B. R. . Conductance-controlled point functionalization of single-walled carbon nanotubes. Science 315, 77–81 (2007). 17204645 10.1126/science.1135303

[b31] Goldsmith B. R., Coroneus J. G., Kane A. A., Weiss G. A. & Collins P. G. Monitoring single-molecule reactivity on a carbon nanotube. Nano Lett. 8, 189–194 (2008). 18088152 10.1021/nl0724079

[b32] Sorgenfrei S. . Label-free single-molecule detection of DNA-hybridization kinetics with a carbon nanotube field-effect transistor. Nature Nanotech. 6, 126–132 (2011). 10.1038/nnano.2010.275 PMC3783941 21258331

[b33] Sorgenfrei S., Chiu C.-Y., Johnston M., Nuckolls C. & Shepard K. L. Debye screening in single-molecule carbon nanotube field-effect sensors. Nano Lett. 11, 3739–3743 (2011). 21806018 10.1021/nl201781q PMC3735439

[b34] Prisbrey L., Roundy D., Blank K., Fifield L. S. & Minot E. D. Electrical characteristics of carbon nanotube devices prepared with single oxidative point defects. J. Phys. Chem. C 116, 1961–1965 (2012).

[b35] Sharf T., Kevek J. W., DeBorde T., Wardini J. L. & Minot E. D. Origins of charge noise in carbon nanotube field-effect transistor biosensors. Nano Lett. 12, 6380–6384 (2012). 23171196 10.1021/nl303651t

[b36] Meir Y. & Wingreen N. S. Landauer formula for the current through an interacting electron region. Phys. Rev. Lett. 68, 2512 (1992). 10045416 10.1103/PhysRevLett.68.2512

[b37] Jauho A., Wingreen N. & Meir Y. Time-dependent transport in interacting and noninteracting resonant-tunneling systems. Phys. Rev. B 50, 5528 (1994). 10.1103/physrevb.50.5528 9976896

[b38] Haug H. & Jauho A.-P. Quantum kinetics in transport and optics of semiconductors (Springer, 1996).

[b39] Reimann S. M. & Manninen M. Electronic structure of quantum dots. Rev. Mod. Phys. 74, 1283 (2002).

[b40] Agrat N., Yeyati A. L. & Van Ruitenbeek J. M. Quantum properties of atomic-sized conductors. Phys. Rep. 377, 81–279 (2003).

[b41] Baruselli P. P., Requist R., Fabrizio M. & Tosatti E. Ferromagnetic Kondo effect in a triple quantum dot system. Phys. Rev. Lett. 111, 047201 (2013). 23931401 10.1103/PhysRevLett.111.047201

[b42] Lake R., Klimeck G., Bowen R. C. & Jovanovic D. Single and multiband modeling of quantum electron transport through layered semiconductor devices. J. Appl. Phys. 81, 7845–7869 (1997).

[b43] Platero G. & Aguado R. Photon-assisted transport in semiconductor nanostructures. Phys. Rep. 395, 1–157 (2004).

[b44] Takei S., Fregoso B. M., Hui H.-Y., Lobos A. M. & Sarma S. D. Soft superconducting gap in semiconductor Majorana nanowires. Phys. Rev. Lett. 110, 186803 (2013). 23683232 10.1103/PhysRevLett.110.186803

[b45] Kramers H. A. Brownian motion in a field of force and the diffusion model of chemical reactions. Physica 7, 284–304 (1940).

[b46] Velizhanin K. A., Sahu S., Chien C.-C., Dubi Y. & Zwolak M. Crossover behavior of the thermal conductance and Kramers’ transition rate theory. *arXiv preprint arXiv:1312.5422* (2013). 10.1038/srep17506 PMC4669443 26634333

[b47] Velizhanin K. A., Sahu S., Chien C.-C., Dubi Y. & Zwolak M. Crossover behavior of the thermal conductance and Kramers’ transition rate theory. Sci. Rep. 5, 17506 (2015). 26634333 10.1038/srep17506 PMC4669443

[b48] Biele R., D’Agosta R. & Rubio A. Time-dependent thermal transport theory. Phys. Rev. Lett. 115, 056801 (2015). 26274434 10.1103/PhysRevLett.115.056801

[b49] Gurvitz S. Rate equations for quantum transport in multidot systems. Phys. Rev. B 57, 6602 (1998).

[b50] Zwolak M. & Di Ventra M. DNA spintronics. Appl. Phys. Lett. 81, 925–927 (2002).

[b51] Ajisaka S., Barra F., Meja-Monasterio C. & Prosen T. Nonequlibrium particle and energy currents in quantum chains connected to mesoscopic Fermi reservoirs. Phys. Rev. B 86, 125111 (2012).

[b52] Ajisaka S. & Barra F. Nonequilibrium mesoscopic Fermi-reservoir distribution and particle current through a coherent quantum system. Phys. Rev. B 87, 195114 (2013).

[b53] Zelovich T., Kronik L. & Hod O. State representation approach for atomistic time-dependent transport calculations in molecular junctions. J. Chem. Theory Comput. 10, 2927–2941 (2014). 26588268 10.1021/ct500135e

[b54] Ajisaka S., Žunkovič B. & Dubi Y. The molecular photo-cell: Quantum transport and energy conversion at strong non-equilibrium. Sci. Rep. 5, 8312 (2015). 25660494 10.1038/srep08312 PMC4321170

[b55] Breuer H.-P. & Petruccione F. The theory of open quantum systems (Oxford university press, 2002).

