# Supplementary material for: Landauer’s formula with finite-time relaxation: Kramers’ crossover in electronic transport
Source: Sci Rep. 2016 Apr 20;6:24514. doi: 10.1038/srep24514 (PMC4837356; doi:10.1038/srep24514)
Supplement: Supplementary Information [file srep24514-s1.pdf]

Landauer's formula with finite-time relaxation: Kramers' crossover in electronic transport – Supplemental Information

Daniel Gruss,[1,2,3] Kirill A. Velizhanin,[4] and Michael Zwolak[1,*]

[1] Center for Nanoscale Science and Technology, National Institute of Standards and Technology, Gaithersburg, MD 20899
[2] Maryland Nanocenter, University of Maryland, College Park, MD 20742
[3] Department of Physics, Oregon State University, Corvallis, OR 97331
[4] Theoretical Division, Los Alamos National Laboratory, Los Alamos, NM 87545

Appendix A: Steady-State Current

The expressions introduced in the main text are based on a derivation of the steady-state current using the equilibrium condition of a single extended reservoir site. What follows in this section is a more complete derivation of the steady-state current.

1. Single Extended Reservoir State Green's Function

Let us a consider a single electronic level of energy $\hbar\omega_k$ connected to a manifold of non-interacting states that comprise the *implicit* reservoir $\mathcal{E}_k$. The index k denotes the extended reservoir state that these states are coupled to. The $\mathcal{E}_{\mathcal{L}(\mathcal{R})}$ from the main text are composed of all $\mathcal{E}_k$ for $k \in \mathcal{L}(\mathcal{R})$. The Hamiltonian of this partial system is

$$H_k = \hbar\omega_k c_k^\dagger c_k + \sum_{\alpha\in\mathcal{E}_k} \hbar\omega_\alpha c_\alpha^\dagger c_\alpha + \sum_{\alpha\in\mathcal{E}_k} \hbar t_\alpha \left(c_\alpha^\dagger c_k + c_k^\dagger c_\alpha \right). \tag{A1}$$

This Hamiltonian describes the systems shown in Fig. 1(b) in the main text. For an implicit reservoir state α, the *isolated* Green's functions are

$$g_\alpha^>(t,t') = -\imath[1-f(\omega_\alpha)]e^{-\imath\omega_\alpha(t-t')-\eta|t-t'|}, \tag{A2}$$

$$g_\alpha^<(t,t') = \imath f(\omega_\alpha)e^{-\imath\omega_\alpha(t-t')-\eta|t-t'|}, \tag{A3}$$

$$g_\alpha^r(t,t') = \theta(t-t')\left[g^>(t,t') - g^<(t,t')\right], \tag{A4}$$

and

$$g_\alpha^a(t,t') = -\theta(t'-t)\left[g^>(t,t') - g^<(t,t')\right]; \tag{A5}$$

or, in terms of their Fourier transforms,

$$g_\alpha^>(\omega) = -2\pi\imath[1-f(\omega_\alpha)]\delta(\omega-\omega_\alpha), \tag{A6}$$

$$g_\alpha^<(\omega) = 2\pi\imath f(\omega_\alpha)\delta(\omega-\omega_\alpha), \tag{A7}$$

$$g_\alpha^r(\omega) = 1/(\omega-\omega_\alpha+\imath\eta), \tag{A8}$$

and

$$g_\alpha^a(\omega) = 1/(\omega-\omega_\alpha-\imath\eta), \tag{A9}$$

where η is the infinitesimal positive number and $f(\omega)$ is the Fermi-Dirac distribution. The subscript α on g_α is used to distinguish it from an extended reservoir state Green's functions (g_k) or the full system Green's functions ($\mathbf{G}_{ij}$) as used for the $\mathcal{L}-\mathcal{S}-\mathcal{R}$ system. For the interaction of one level with all the other levels, Eq. (A1), we have (symbolically, on the Keldysh contour) $g_k = g_{0k} + g_{0k}\Sigma_k g_k$, with g_{0k} being the single isolated *extended* reservoir site. Using the relation between on-contour and real-time non-equilibrium Green's functions,[1] for the retarded Green's function we have

$$g_k^r(\omega) = g_{0k}^r(\omega) + g_{0k}^r(\omega)\Sigma_k^r(\omega)g_k^r(\omega), \tag{A10}$$

where

$$\Sigma_k^r(\omega) = \sum_{\alpha\in\mathcal{E}_k} t_\alpha^2 g_\alpha^r(\omega) = \frac{1}{2\pi}\int d\omega' \frac{\gamma(\omega')}{\omega-\omega'+\imath\eta}. \tag{A11}$$

Here, $\gamma(\omega) = 2\pi\sum_\alpha t_\alpha^2\delta(\omega-\omega_\alpha)$. Evaluating the integral, one obtains

$$\Sigma_k^r(\omega) = \frac{1}{2\pi}\mathcal{P}\int d\omega' \frac{\gamma(\omega')}{\omega-\omega'} - \frac{\imath\gamma(\omega)}{2} = E_k(\omega) - \frac{\imath\gamma(\omega)}{2}, \tag{A12}$$

where $E_k(\omega)$ is a frequency-dependent energy shift and $\gamma(\omega)$ is a frequency-dependent relaxation rate. Similarly, the lesser self-energy is evaluated as

$$\Sigma_k^<(\omega) = \sum_{\alpha\in\mathcal{E}_k} t_\alpha^2 g_\alpha^<(\omega) = \imath\gamma(\omega)f(\omega). \tag{A13}$$

The retarded Green's function of the extended reservoir site then becomes

$$g_k^r(\omega) = \frac{1}{\omega - [\omega_k + E_k(\omega)] + \imath\gamma(\omega)/2}. \tag{A14}$$

As is seen, "friction" is in general non-Markovian, since the dephasing rate $\gamma(\omega)$ is frequency-dependent. The frequency shift, $E_k(\omega)$, is also non-Markovian in the same sense. Now let us find the the full lesser Green's function. To this end we will use the Keldysh equation[1,2]

$$g_k^<(\omega) = g_k^r(\omega)\Sigma_k^<(\omega)g_k^a(\omega), \tag{A15}$$

resulting in

$$g_k^<(\omega) = \frac{\imath\gamma(\omega)f(\omega)}{(\omega-\omega_k-E_k(\omega))^2 + \gamma^2(\omega)/4}. \tag{A16}$$

The important fact here is that the Fermi-Dirac distribution here is not evaluated at ω_k or at any other fixed frequency. Instead, it is evaluated at ω and, therefore, this factor is the same (at fixed ω) for any Green's function of any site within the same reservoir. In particular, it guarantees that the current vanishes when the Fermi-Dirac distribution is the same for the two extended reservoirs.

The often used wide band approximation would result in ω-independent γ and vanishing E_k. In this approximation, the result for the lesser Green's function is

$$g_k^<(\omega) = \frac{\imath\gamma f(\omega)}{(\omega-\omega_k)^2+\gamma^2/4}. \quad (A17)$$

In a similar manner, the retarded Green's function is

$$g_k^r(\omega) = \frac{1}{\omega-\omega_k+\imath\gamma/2}. \quad (A18)$$

Accordingly, one has, using the identity $g_k^r(\omega) = [g_k^a(\omega)]^*$,

$$g_k^<(\omega) = -f(\omega)\left[g_k^r(\omega) - g_k^a(\omega)\right]. \quad (A19)$$

2. Landauer-like Formula

We are following a notation similar to the original Meir-Wingreen paper.[3] The Hamiltonian of the system is

$$H = \sum_{k\in\mathcal{L},\mathcal{R}} \epsilon_k c_k^\dagger c_k + \sum_{k\in\mathcal{L},\mathcal{R}}\sum_{i\in\mathcal{S}} \hbar\left(v_{ki}c_k^\dagger c_i + v_{ik}c_i^\dagger c_k\right) + H_{\mathcal{S}}, \quad (A20)$$

with $\epsilon_k = \hbar\omega_k$. Unlike the Meir-Wingreen scenario where such a Hamiltonian fully describes the steady-state since the sizes of left and right reservoirs are assumed to be infinite, here we take a finite number of extended reservoir states, but each such site k is connected to an implicit reservoir according to the Hamiltonian (A1).

The time-dependent current from the left reservoir to the system can be written as[3]

$$I(t) = e\sum_{k\in\mathcal{L}}\sum_{j\in\mathcal{S}}\left[v_{kj}\mathbf{G}_{jk}^<(t,t) - v_{jk}\mathbf{G}_{kj}^<(t,t)\right]. \quad (A21)$$

When the steady state is established, we can take the Fourier transform,

$$I = e\sum_{k\in\mathcal{L}}\sum_{j\in\mathcal{S}}\int\frac{d\omega}{2\pi}\left[v_{kj}\mathbf{G}_{jk}^<(\omega) - v_{jk}\mathbf{G}_{kj}^<(\omega)\right]. \quad (A22)$$

Since the reservoir sites are non-interacting (in a two- or more-electron sense) we have the following Dyson equation[2]

$$G_{kj}^<(\omega) = \sum_{i\in\mathcal{S}} v_{ki}\left[g_k^r(\omega)\mathbf{G}_{ij}^<(\omega) + g_k^<(\omega)\mathbf{G}_{ij}^a(\omega)\right], \quad (A23)$$

or equivalently

$$G_{ik}^<(\omega) = \sum_{j\in qs} v_{jk}\left[\mathbf{G}_{ij}^r(\omega)g_k^<(\omega) + \mathbf{G}_{ij}^<(\omega)g_k^a(\omega)\right]. \quad (A24)$$

Using these identities, Eq. (A22) can be rewritten as

$$I = e\sum_{k\in\mathcal{L}}\sum_{i,j\in\mathcal{S}}\int\frac{d\omega}{2\pi}v_{ki}v_{jk}\left\{g_k^<(\omega)\left[\mathbf{G}_{ij}^r(\omega) - \mathbf{G}_{ij}^a(\omega)\right] - \left[g_k^r(\omega) - g_k^a(\omega)\right]\mathbf{G}_{ij}^<(\omega)\right\}. \quad (A25)$$

We emphasize that even though $g_k(\omega)$ are single-particle non-interacting Green's functions, the corresponding quasiparticles do have finite lifetime because of their coupling to the implicit reservoirs, which is different from the original Meir-Wingreen formulation. For example, $g_k^{\gtrless}(\omega)$ are not delta functions with respect to ω, but rather Lorentzians (when the wide band limit is taken, Eq. (A17)).

To proceed further we use (i) the Keldysh equation,[1,2] $\mathbf{G}^{\gtrless} = \mathbf{G}^r\mathbf{\Sigma}^{\gtrless}\mathbf{G}^a$, and (ii) that the self-energy due to the interaction with reservoir sites is $\mathbf{\Sigma}_{ij}^{r(a)} = \sum_{k\in\mathcal{L},\mathcal{R}} v_{ik}v_{kj}g_k^{r(a)}$, and (iii) the linear relation between real-time Green's functions $\mathbf{G}^> - \mathbf{G}^< = \mathbf{G}^r - \mathbf{G}^a$.[1] Using these identities the current can be rewritten as

$$I = e\sum_{i,j\in\mathcal{S}}\sum_{\alpha,\beta\in\mathcal{S}}\sum_{k\in\mathcal{L}}\sum_{l\in\mathcal{L},\mathcal{R}}\int\frac{d\omega}{2\pi}v_{ki}v_{jk}v_{\alpha l}v_{l\beta}\mathbf{G}_{i\alpha}^r(\omega)\mathbf{G}_{\beta j}^a(\omega)\left\{g_k^<(\omega)\left[g_l^r(\omega) - g_l^a(\omega)\right] - \left[g_k^r(\omega) - g_k^a(\omega)\right]g_l^<(\omega)\right\}. \quad (A26)$$

Considering the equilibrium property of the isolated state k, Eq. (A19), one gets

$$I = -e\sum_{i,j\in\mathcal{S}}\sum_{\alpha,\beta\in\mathcal{S}}\sum_{k\in\mathcal{L}}\sum_{l\in\mathcal{R}}\int\frac{d\omega}{2\pi}v_{ki}v_{jk}v_{\alpha l}v_{l\beta}\mathbf{G}_{i\alpha}^r(\omega)\mathbf{G}_{\beta j}^a(\omega)\left[g_l^r(\omega) - g_l^a(\omega)\right]\left[g_k^r(\omega) - g_k^a(\omega)\right]\left\{f_{\mathcal{L}}(\omega) - f_{\mathcal{R}}(\omega)\right\}. \quad (A27)$$

It is clearly seen that once the Fermi-Dirac distribution becomes identical on the left and on the right, the current

vanishes. Actually, any partial current also vanishes, i.e., the current for a specific choice of indices $m, n, \alpha, \beta, k, l$ and frequency ω, as of course is expected due to the necessary detailed balance at equilibrium.

A concise expression for the current can be written by introducing the spectral density

$$\mathbf{\Gamma}^{\mathcal{L}(\mathcal{R})}_{ji}(\omega) = \imath \sum_{k\in\mathcal{L}(\mathcal{R})} v_{jk} v_{ki} \left[g^r_k(\omega) - g^a_k(\omega)\right], \tag{A28}$$

and the expression for the current becomes

$$I = \frac{e}{2\pi} \sum_{i,j,\alpha,\beta\in S} \int_{-\infty}^{\infty} d\omega \; [f_{\mathcal{L}}(\omega) - f_{\mathcal{R}}(\omega)] \times \mathbf{\Gamma}^{\mathcal{L}}_{ij}(\omega)\mathbf{G}^r_{j\alpha}(\omega)\mathbf{\Gamma}^{\mathcal{R}}_{\alpha\beta}(\omega)\mathbf{G}^a_{\beta i}(\omega). \tag{A29}$$

Using the matrix notation,

$$I = \frac{e}{2\pi} \int_{-\infty}^{\infty} d\omega \; [f_{\mathcal{L}}(\omega) - f_{\mathcal{R}}(\omega)] \times \mathrm{tr}\left[\mathbf{\Gamma}^{\mathcal{L}}(\omega)\mathbf{G}^r(\omega)\mathbf{\Gamma}^{\mathcal{R}}(\omega)\mathbf{G}^a(\omega)\right]. \tag{A30}$$

This expression is Eq. (3) from the main text.

3. Small γ, $N_r \to \infty$

As an example, we examine a system that is a single noninteracting state. In this case, the continuous form for the extended reservoir self-energy from integrating the single site Green's functions, Eq. (12) from the main text, is

$$\Sigma^{r(a)}(\omega) = \left(\omega \mp \imath\gamma/2 - \imath\sqrt{4J^2 - (\omega \mp \imath\gamma/2)^2}\right)/2, \tag{A31}$$

when $W = 4J$, and can be broken into the real and imaginary components

$$\mathrm{Re}\,\Sigma^r(\omega) = \frac{1}{4}\left[8\left(\omega^2 + 4J^2\right)\gamma^2 + 16\left(\omega^2 - 4J^2\right)^2 + \omega^4\right]^{\frac{1}{4}} \times \sin\left[\frac{1}{2}\tan^{-1}\left(\frac{-4\omega\gamma}{-4\omega^2 + \gamma^2 + 16J^2}\right)\right] + \frac{\omega}{2} \tag{A32}$$

$$\mathrm{Im}\,\Sigma^r(\omega) = -\frac{1}{2}\left[\left(\omega^2 - \frac{\gamma^2}{4} - 4J^2\right)^2 + \omega^2\gamma^2\right]^{\frac{1}{4}} \times \cos\left[\frac{1}{2}\tan^{-1}\left(\frac{-4\omega\gamma}{-4\omega^2 + \gamma^2 + 16J^2}\right)\right] - \frac{\gamma}{4}. \tag{A33}$$

The term in the integral of Eq. (A30) can then be written in terms of these components, and then expanded in γ and ω. Since both γ and ω appear as the same order in the expansion and the self-energy is small outside the band edge, the error in the ω integral is small when γ is small. In practice, the bias is typically also taken to be small. For a single central site,

$$\begin{aligned} T_\gamma(\omega) &= \mathrm{tr}\left[\mathbf{\Gamma}^{\mathcal{L}}(\omega)\mathbf{G}^r(\omega)\mathbf{\Gamma}^{\mathcal{R}}(\omega)\mathbf{G}^a(\omega)\right] \\ &= \frac{(2\,\mathrm{Im}\,\Sigma^r(\omega))^2}{(\omega - 2\,\mathrm{Re}\,\Sigma^r(\omega))^2 + (2\,\mathrm{Im}\,\Sigma^r(\omega))^2} \approx 1 - \frac{\omega^2\gamma^2}{64J^4}. \end{aligned} \tag{A34}$$

The first term is the transmission coefficient for a 1D lattice in the Landauer formula, so the correction is of order γ^2.

4. Large γ

As γ increases, the Green's function for a single reservoir site, Eq. (A18), approaches $g^r_k(\omega) = -2/\gamma$ in the relevant region of integration (i.e., where the difference in Fermi distributions is non-negligible). The transmission coefficient in that region, then, is

$$T_\gamma(\omega) \approx -\frac{4}{\gamma}\sum_k v_k^2\,\mathrm{Im}[\mathbf{G}^r(\omega)]. \tag{A35}$$

Using the explicit form for the total Green's function, with $\mathcal{S}$ consisting of a single non-interacting state with frequency ω_s, this simplifies to

$$\begin{aligned} T_\gamma(\omega) &\approx \frac{4}{\gamma}\sum_k v_k^2\left(\frac{4\sum_k v_k^2/\gamma}{(\omega-\omega_s)^2 + (4\sum_k v_k^2/\gamma)^2}\right) \\ &= \frac{1}{\gamma}\left(\frac{1/\gamma}{(\omega-\omega_s)^2/(4J^2)^2 + (1/\gamma)^2}\right) \\ &\approx \frac{\pi}{\gamma}\delta\left(\frac{\omega-\omega_s}{4J^2}\right) = \frac{4\pi J^2}{\gamma}\delta(\omega-\omega_s), \end{aligned} \tag{A36}$$

where we used that $\sum_k v_k^2 = J^2$ since the couplings v_k come from a unitary transformation times the total coupling to the system. When the bias window includes this peak and at zero temperature, the current in the large γ regime becomes

$$I_3 \approx \frac{2eJ^2}{\gamma}. \tag{A37}$$

This expression is independent of the bias, as long as it is nonzero. As we note in the main text, the transition to this value of the current, though, does depend on the bias.

Appendix B: Markovian Master Equation

In the main text and above, we derived a Landauer formula for the $\mathcal{L}-\mathcal{S}-\mathcal{R}$ system, which results in a Markovian master equation for the real-time dynamics in the small-to-intermediate γ regime. As we show below and mention in the main text, outside of this regime this formalism gives physically invalid results. In the appropriate region, though, it allows for the direct calculation of

the full time dynamics and can be readily expanded to include many-body interactions or time-dependent terms. Here we will derive the full solution—in all regimes—to the Markovian master equation. Given the Hamiltonian

$$H = \sum_{k\in\mathcal{L},\mathcal{R}} \epsilon_k c_k^\dagger c_k + \sum_{k\in\mathcal{L},\mathcal{R}} \sum_{i\in\mathcal{S}} \hbar \left(v_{ki} c_k^\dagger c_i + v_{ik} c_i^\dagger c_k \right) + H_{\mathcal{S}}, \tag{B1}$$

the starting point is the Markovian master equation,

$$\dot\rho = -\frac{\imath}{\hbar}\left[H,\rho\right] + \sum_k \gamma_{k^+} \left(c_k^\dagger \rho c_k - \frac{1}{2}\left\{ c_k c_k^\dagger, \rho \right\} \right) + \sum_k \gamma_{k^-} \left(c_k \rho c_k^\dagger - \frac{1}{2}\left\{ c_k^\dagger c_k, \rho \right\} \right), \tag{B2}$$

with $\gamma_{k^+} = \gamma f_k^\alpha$ and $\gamma_{k^-} = \gamma(1 - f_k^\alpha)$, where $\alpha = \mathcal{L}(\mathcal{R})$ when $k \in \mathcal{L}(\mathcal{R})$, and $f_k^{\mathcal{L}(\mathcal{R})} = 1/(\exp[\beta(\epsilon_k - V_{\mathcal{L}(\mathcal{R})})] + 1)$ is the Fermi-Dirac distribution. The total applied bias is $V = V_\mathcal{L} - V_\mathcal{R}$. This form for γ_{k^+} and γ_{k^-} ensures that in the absence of the interaction $H_\mathcal{I}$, the extended reservoirs will relax into an independent equilibrium of their own Hamiltonians, $H_\mathcal{L}$ and $H_\mathcal{R}$. For simplicity, we have assumed that γ is the same in both the left and right regions, and for all k.

This master equation describes the evolution of a system in the presence of explicit reservoir states, with a different mechanism than used in the main text. The following will examine the behavior of Eq. (B2) over the full range of the relaxation γ.

1. Single Extended Reservoir State Green's Function

In the absence of $\mathcal{S}$, the extended reservoir states decay into the equilibrium state, $\exp(-\beta H_{\mathcal{L}(\mathcal{R})})/Z$, i.e., the occupations decay to a Fermi-Dirac distribution as $e^{-\gamma t/2}$. This can be shown from the exact time-dependent lesser Green's function in a reservoir site uncoupled from $\mathcal{S}$, which is given by

$$g_k^<(0,t) = \imath \left\langle c_k^\dagger(t) c_k(0) \right\rangle = \imath \mathrm{tr}\left[c_k^\dagger e^{Lt} c_k \rho_{eq} \right], \tag{B3}$$

where $L\rho = d\rho/dt$ can be found from Eq. (B2) and the superoperator L is the Lindbladian. The equilibrium state of the site has filling f_k, so $\mathrm{tr}\left[c_k^\dagger c_k \rho_{eq} \right] = f_k$. In the Fock basis of a single state, the equilibrium state is

$$\rho_{eq} = \begin{pmatrix} 1 - f_k & 0 \\ 0 & f_k \end{pmatrix}. \tag{B4}$$

A Jordan-Wigner transformation maps the electron creation and annihilation operators onto spin operators, which allows us to write $c_k \rho_{eq} = (f_k \sigma_x + \imath f_k \sigma_y)/2$. For the equation of motion, Eq. (B2), the Lindblad operator is block diagonal in the the σ_x, σ_y subspace and σ_I, σ_z subspace. This means we can separately solve for the dynamics using L for these two subspaces. If we wish to calculate the action on a generic operator $O = a_0\sigma_I + a_x\sigma_x + a_y\sigma_y + a_z\sigma_z$, for the σ_x, σ_y subspace:

$$\sigma_x \frac{da_x}{dt} + \sigma_y \frac{da_y}{dt} = -\imath[\omega_k c_k^\dagger c_k, a_x\sigma_x + a_y\sigma_y] + \gamma_{k^+} \left(c_k^\dagger (a_x\sigma_x + a_y\sigma_y) c_k - \frac{1}{2}\{ c_k^\dagger c_k, a_x\sigma_x + a_y\sigma_y \} \right) + \gamma_{k^-} \left(c_k (a_x\sigma_x + a_y\sigma_y) c_k^\dagger - \frac{1}{2}\{ c_k c_k^\dagger, a_x\sigma_x + a_y\sigma_y \} \right). \tag{B5}$$

Acting on both sides with $(1/2)\sigma_x \mathrm{tr}$ gives $da_x/dt = -\gamma a_x/2 + \omega_k a_y$ with $\gamma = \gamma_{k^+} + \gamma_{k^-}$. Similarly with $(1/2)\sigma_y \mathrm{tr}$, giving $da_y/dt = -\gamma a_y/2 - \omega_k a_x$. Solving these equations of motion we obtain

$$e^{Lt} c_k \rho_{eq} = \sigma_x f_k/2 e^{-t\gamma/2 + \imath\omega_k t} + \sigma_y f_k/2 e^{-t\gamma/2 - \imath\omega_k t}. \tag{B6}$$

Then acting with c_k and taking the trace gives

$$g_k^<(0,t) = \imath e^{-t\gamma/2 + \imath\omega_k t} f_k, \tag{B7}$$

for $t \geq 0$. This can be readily employed to find the retarded and advanced Green's functions for the single state

$$g_k^{r(a)}(t,t') = \mp\imath\Theta(\pm t \mp t') e^{-\imath\omega_k(t-t') - \gamma|t-t'|/2}. \tag{B8}$$

or its Fourier transform, $g_k^{r(a)}(\omega) = (\omega - \omega_k \pm \imath\gamma/2)^{-1}$. Physically, the reservoir sites are exchanging electrons with a larger external reservoir with an infinite number of electrons and states without memory. The lesser Green's function is also found to be

$$g_k^<(\omega) = -f_k\left[g_k^r(\omega) - g_k^a(\omega) \right]. = \frac{\imath\gamma f(\omega_k)}{(\omega - \omega_k)^2 + \gamma^2/4}. \tag{B9}$$

This expression recovers the original lesser Green's function, Eq. (A17), except that the distribution is evaluated at the ω_k of the state, rather than being a continuum over ω. As we discuss below, this has the effect of broadening the density of states after they are occupied, rather than occupying after broadening.

2. Steady-State Current

The general solution to the steady-states of the master equation (Eq. (B2)) can also be found in an analogous way to Jauho, Meir, and Wingreen and follows the same process as the derivation for Eq. (A30). In this case we use the equilibrium relation from the previous section, Eq. (B9), rather than using the one that has an ω-dependent distribution $f(\omega)$. In practice, this derivation is the same but with a filling, f_k, dependent on the reservoir energy, ϵ_k.

That is, after applying the Markovian equilibrium property, Eq. (B9), the expression for the current, Eq. (A27), is instead

$$I = -e\sum_{i,j\in S}\sum_{\alpha,\beta\in S}\sum_{k\in L}\sum_{l\in R}\int\frac{d\omega}{2\pi}v_{ki}v_{jk}v_{\alpha l}v_{l\beta}\mathbf{G}^r_{i\alpha}(\omega)\mathbf{G}^a_{\beta j}(\omega)\left[g^r_l(\omega)-g^a_l(\omega)\right]\left[g^r_k(\omega)-g^a_k(\omega)\right]\{f_k-f_l\}\,. \tag{B10}$$

Again, a concise expression for the current can be written by introducing the spectral density

$$\mathbf{\Gamma}^{\mathcal{L}(\mathcal{R})}_{ji}(\omega) = \imath\sum_{k\in\mathcal{L}(\mathcal{R})}v_{jk}v_{ki}\left[g^r_k(\omega)-g^a_k(\omega)\right], \tag{B11}$$

and the population-weighted spectral density

$$\tilde{\mathbf{\Gamma}}^{\mathcal{L}(\mathcal{R})}_{ji}(\omega) = \imath\sum_{k\in\mathcal{L}(\mathcal{R})}f_k v_{jk}v_{ki}\left[g^r_k(\omega)-g^a_k(\omega)\right], \tag{B12}$$

where the Fermi-Dirac distributions are included. Then, the current can be written as

$$I = \frac{e}{2\pi}\int_{-\infty}^{\infty}d\omega\ \mathrm{tr}\Big[\tilde{\mathbf{\Gamma}}^{\mathcal{L}}(\omega)\mathbf{G}^r(\omega)\mathbf{\Gamma}^{\mathcal{R}}(\omega)\mathbf{G}^a(\omega) - \mathbf{\Gamma}^{\mathcal{L}}(\omega)\mathbf{G}^r(\omega)\tilde{\mathbf{\Gamma}}^{\mathcal{R}}(\omega)\mathbf{G}^a(\omega)\Big], \tag{B13}$$

Note that, in this case, the integrand in Eq. (B13) does not include the Fermi-Dirac distribution as a separate prefactor as in the Meir-Wingreen / Landauer formula, but rather appears as a convolution with a Lorentzian due to the inclusion of a finite relaxation time from the Markovian master equation.

When the reservoir states are symmetrically coupled to the system, a simplified expression results:

$$I = -\frac{e}{2\pi}\sum_{k\in\mathcal{L}}\sum_{i,j\in\mathcal{S}}v_{ik}v_{kj}(f^{\mathcal{L}}_k - f^{\mathcal{R}}_k) \times\int_{-\infty}^{\infty}\frac{d\omega\ \gamma}{(\omega-\omega_k)^2+\gamma^2/4}\mathrm{Im}[\mathbf{G}^r_{ij}(\omega)]. \tag{B14}$$

For this symmetric case, when the two extended reservoirs have the same chemical potential, the calculated current I is always equal to zero. However, in the asymmetric case, Eq. (B10) can yield a non-zero current even with no applied bias. We will examine this fact in Appendix C in order to develop a bound for when the Markovian master equation is consistent with physical expectations.

3. Small and Large γ Limits

In similar fashion to the main text, we can derive the limiting expressions for the steady-state current in the low and high relaxation rate regimes. The spectral function for a single reservoir site connected to an implicit bath is given by

$$A_k(\omega) = \imath\left[g^r_k(\omega)-g^a_k(\omega)\right] = \frac{\gamma}{(\omega-\omega_k)^2+\gamma^2/4}. \tag{B15}$$

If γ is very small, $A_k(\omega)$ approaches $2\pi\delta(\omega-\omega_k)$. Then $A_k(\omega)f_k \approx A_k(\omega)f_{\mathcal{L}(\mathcal{R})}(\omega)$, so long as $f(\omega)$ changes little over the width of $A_k(\omega)$, and the expression for the current can be rewritten as

$$I_1 \approx \frac{e}{2\pi}\int_{-\infty}^{\infty}d\omega\ \left[f_{\mathcal{L}}(\omega)-f_{\mathcal{R}}(\omega)\right] \times \mathrm{tr}\left[\mathbf{\Gamma}^{\mathcal{L}}(\omega)\mathbf{G}^r(\omega)\mathbf{\Gamma}^{\mathcal{R}}(\omega)\mathbf{G}^a(\omega)\right]. \tag{B16}$$

This recovers Eq. (3) from the main text, where the spectral density and Green's functions include the relaxation rate γ. What this means is that the master equation can be used to simulate transport, provided that γ is in a suitable range. Even the intermediate regime can be accurately simulated, so long as N_r is sufficiently large.

Similarly for large γ, the integral within Eq. (B14) can be calculated by transforming out from the frequency domain:

$$\int_{-\infty}^{\infty}\frac{d\omega\ \gamma}{(\omega-\omega_k)^2+\gamma^2/4}\mathbf{G}^r_{ij}(\omega) = 2\pi\int_{-\infty}^{\infty}dt\, e^{-\imath\omega_k-\gamma|t|/2}\ \mathbf{G}^r_{ij}(-t) \approx -\imath\frac{4\pi}{\gamma}\delta_{i,j}, \tag{B17}$$

which applies to both interacting and non-interacting Green's functions. In the non-interacting case, this can be thought of an integration over the density of states for each site in $\mathcal{S}$. This yields an expression for the current in the large γ regime to be

$$I_3 \approx 2e/\gamma\sum_{k\in\mathcal{L}}\sum_{i\in\mathcal{S}}v^2_{ki}(f^{\mathcal{L}}_k - f^{\mathcal{R}}_k), \tag{B18}$$

where again the sum is over just a single set of k (either in the left or right extended reservoir, which are identical).

When $\mathcal{S}$ consists of a single site, then the large-γ current is found to be

$$I_3 \approx 2e/\gamma\sum_{k\in\mathcal{L}}v^2_k(f^{\mathcal{L}}_k - f^{\mathcal{R}}_k). \tag{B19}$$

The sum of the v^2_k terms is the transformation that diagonalizes the extended reservoir's single particle Hamiltonian.[4] Thus,

$$\sum_{k\in\mathcal{L}(\mathcal{R})}f^{\mathcal{L}(\mathcal{R})}_k v^2_k/J^2 \to n^{\mathcal{L}(\mathcal{R})}, \tag{B20}$$

where $n^{\mathcal{L}(\mathcal{R})}$ is the occupation of the extended reservoir state in real space at the site immediately adjacent to the system on the left (right). This makes a correspondence with a setup with just a single extended reservoir site on each end ($N_r = 1$), $I_3 \approx 2eJ^2(n^{\mathcal{L}} - n^{\mathcal{R}})/\gamma$. Essentially, the sites further away in the $N_r > 1$ case are effectively

decoupled from the system as any flow of electrons away from those sites is suppressed due to the strong relaxation. Therefore, the current in the large γ regime is also independent of N_r with the exception of discretization effects.

In contrast to the Landauer-like formalism, the Markovian master equation allows for an explicit derivation showing only the occupation at the boundaries matter, i.e., all other electrons are prevented from flowing to the system by the strong relaxation. This is possible due to the well-defined occupation of each reservoir site. In the full model, the states are broadened and then occupied, so there is no equivalent transformation to a single edge site occupation like there is in the Markovian case.

We can, however, go further with the Markovian procedure. Within a small bias window, the reservoir coupling is approximately constant $v_k^2 \approx J^2/N_r$, and the sum of the occupation terms is approximately the total number of states within the bias window $\sum_k (f_k^{\mathcal{L}} - f_k^{\mathcal{R}}) \approx VN_r/(W\hbar)$. Substituting this in, we find $I_3 \approx eV[2\pi J^2/(\gamma W)](2\pi\hbar)$. Comparing with Eq. (10) in the main text, the large γ current here is additionally inversely related to the bandwidth and grows linearly with V. Thus, while there is similar physical behavior to the full model, the large γ current is quantitatively very different for the Markovian master equation, which reflects its lack of validity in this regime.

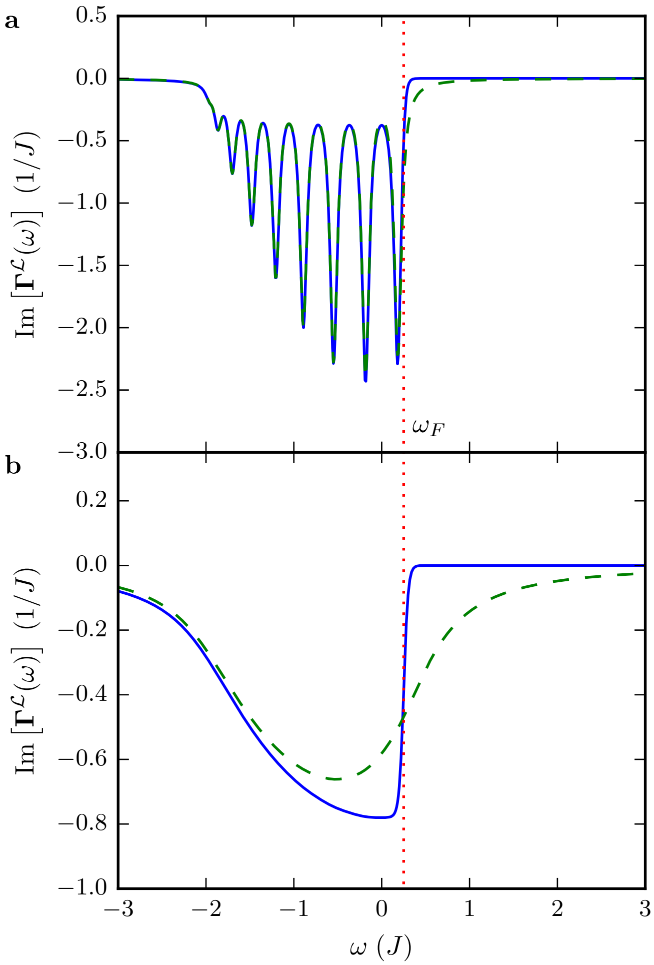

Figure 1. **Spectral density and population-weighted spectral density for a 1D reservoir.** The solid line is the spectral density, Eq. (A28) or Eq. (B11), times the Fermi-Dirac distribution and the dashed line is the population-weighted spectral density, Eq. (B12). These correspond to the Landauer-like formula and the Markovian master equation method respectively. The number of reservoir states $N_r = 16$, bias and temperature are the same as the main text, $\beta = 40(J\hbar)^{-1}$ and $\omega_F = 0.25J$, and the relaxation rate is (**a**) $\gamma = 0.1J$ and (**b**) $\gamma = J$.

Appendix C: Bounds on the Validity of the Markovian Master Equation

This section quantifies the regimes where the master equation is physically valid. The Markovian master equation is always mathematically valid in that it gives proper quantum evolution. However, as we will see, it does not accurately represent the equilibrium state at larger values of γ, which, e.g., leads to spurious currents and a break down of detailed balance.

1. Broadening and the Fermi Level

The Markovian master equation broadens extended reservoir states across a wide range of ω when the relaxation rate γ is large. That is, even with the Fermi level fixed in the isolated extended reservoir, there is excessive electron occupation beyond this level in the open system (succinctly, the Markovian equation occupies the states then broadens them, rather than broadening then occupying). As an example, Fig. 1 shows the density of states times the occupation for both the full and Markovian approaches. For small γ, Fig. 1(a), the relaxation is weak enough that the states are still relatively localized in energy. However, for large γ, Fig. 1(b), the electronic occupation is smeared too much, and this allows current to flow even without a drop in chemical potential. This difference is most apparent for states at the Fermi level, $\omega_k \approx \omega_F$, as this is where the distribution is most rapidly changing.

In the two approaches, only the lesser Green's functions are different, and these only differ by the distribution function, $f(\omega)$ compared to $f(\omega_k)$. We quantify the error, Δ, between the two by the integrated absolute difference,

$$\Delta = \int_{-\infty}^{\infty} d\omega \frac{\gamma\,|f(\omega) - f(\omega_k)|}{(\omega - \omega_k)^2 + \gamma^2/4}. \qquad \text{(C1)}$$

To upper bound this error, we will use two features of Δ. One is that the error is maximal when the state ω_k is at the Fermi level, $\omega_k = \omega_F$. Two is that the Fermi distribution can be replaced by the piecewise continuous

function

$$f(\omega) \to \begin{cases} 1, & \omega \le \omega_F - \frac{2}{\beta\hbar} \\ \frac{1}{2} - \frac{1}{4}\beta\hbar(\omega - \omega_F), & |\omega - \omega_F| < \frac{2}{\beta\hbar} \\ 0, & \omega \ge \omega_F + \frac{2}{\beta\hbar} \end{cases} \quad \text{(C2)}$$

in order to obtain a bound of the error at the Fermi level. That is, this replacement has a greater absolute difference to $f(\omega_F) = 1/2$ than the original distribution function for all ω. Using these two features, the error for any ω_k is bounded by

$$\Delta \le \arctan\left(\frac{-4}{\gamma\beta\hbar}\right) + \frac{\pi}{2} + \frac{\gamma\beta\hbar}{4}\ln\left(1 + \frac{16}{\gamma\beta\hbar}\right) \quad \text{(C3)}$$
$$\lessapprox \frac{\gamma\beta\hbar}{4}\ln\left(\frac{1}{\gamma\beta\hbar}\right).$$

The condition for this error to be small is then $\gamma\beta\hbar \ll 1$. This can be interpreted as requiring that the broadening due to the relaxation must be smaller than the broadening caused by thermal processes. As well, it has a simple, intuitive mathematical meaning: The maximal slope of the Fermi-Dirac distribution should be much smaller than γ, so that the γ-induced smearing has no significant effect on the occupation.

Expanding on the above brief account, we can show that the error is maximal when ω_k is at the Fermi level by extremizing Δ,

$$\frac{d\Delta}{d\omega_k} = \frac{d}{d\omega_k}\int_{-\infty}^{\infty} d\omega \frac{\gamma\,|f(\omega) - f(\omega_k)|}{(\omega - \omega_k)^2 + \gamma^2/4} = 0, \quad \text{(C4)}$$

which can be rewritten as

$$\int_{-\infty}^{\infty} d\omega \frac{\gamma\,\mathrm{sign}(\omega_k - \omega)(f'(\omega) - f'(\omega_k))}{(\omega - \omega_k)^2 + \gamma^2/4} = 0. \quad \text{(C5)}$$

When ω_k is at the Fermi level, the term containing $f'(\omega_k)$ integrates to zero, as it is a symmetric function multiplied by an antisymmetric function around ω_k. Also when ω_k is at the Fermi level, $f'(\omega)$ is symmetric around ω_F (which comes from the relationship $f(\omega_F - \omega) = 1 - f(\omega_F + \omega)$ when $\omega_k = \omega_F$), so $f'(\omega_F - \omega) = f'(\omega_F + \omega)$. Thus, the integrand is antisymmetric and it evaluates to zero. This therefore gives an extremum in the error. Moreover, when $\omega_k < \omega_F$, the slope is positive and when $\omega_k > \omega_F$, the slope is negative, therefore the error is maximal at the Fermi level. In the limiting cases, $\omega_k \to \pm\infty$, the total error Δ is zero, meaning that there is agreement when the state is far away from the Fermi level, as excess smearing has no consequences when the distribution is flat.

Additionally, bounds on the relaxation rate and the number of states required can be found for a given system by numerically integrating the spectral density above the Fermi level for the zero temperature case. This yields a direct measure of the improperly occupied high-energy states, even when they do not directly contribute to the electronic current.

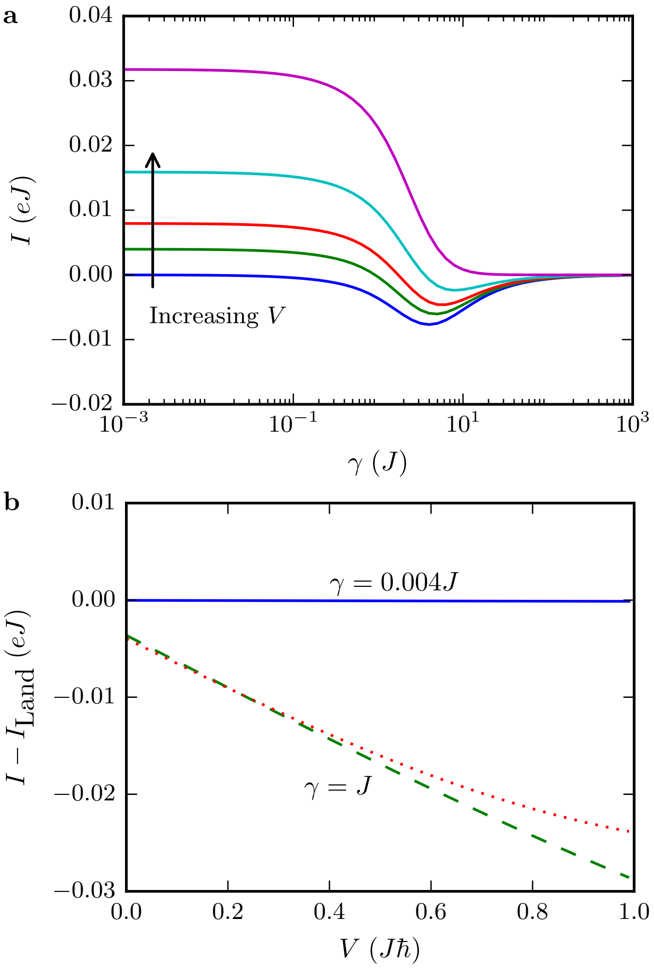

Figure 2. **Asymmetric system response.** (**a**) V the bias dependence of current I versus γ. The bias is $V \in \{0, 0.025, 0.05, 0.1, 0.2\}J\hbar$ from bottom to top. The reservoirs states are equally spaced with a bandwidth of $4J$, $\beta = 40(J\hbar)^{-1}$, and the left reservoir is shifted by $\delta_{\mathcal{L}} = 0.1J$ and the right is $\delta_{\mathcal{R}} = -0.1J$, making $\delta = 0.2J$. Note there is a negative current for no applied bias. (**b**) Difference between the current I and the Landauer calculation I_{Land} versus the applied bias V for $N_r = \infty$. The solid line is near the peak current, $\gamma = 0.004J$, and the dashed line is $\gamma = J$. The dotted line is the approximation from low temperature and high bandwidth Eq. (C7) for $\gamma = J$.

2. Asymmetric Reservoirs

Next, we will examine a system in between asymmetric reservoirs. When the asymmetry of the $\mathcal{L}$ and $\mathcal{R}$ extended reservoir is due to a shift in their relative energies by δ, then we have $\omega'_k = \omega_k + \delta/2$ for $k \in \mathcal{L}$ and $\omega'_k = \omega_k - \delta/2$ for $k \in \mathcal{R}$. Solving the equation of motion, Eq. (B2), shows that there is a non-zero current for the zero applied bias ($V = 0$). Note that δ is a parameter that quantifies the asymmetry of the system.

Physically, the Fermi level on both reservoirs are identical, so the steady-state current between them should be

zero. When the reservoir density of states is broadened by γ, the replacement of $f(\omega)$ by $f(\omega_k)$, as the Markovian model does, results in some electronic occupation above the Fermi level. This can give rise to an electronic current. Figure 2 shows the steady-state current I as a function of the relaxation γ for increasing values of the applied bias V.

To simplify the analytic forms for the steady-state current, we will introduce a system where the extended reservoirs are "Markovian" rather than 1D. The density of states for the 1D case is approximately constant near $\omega = 0$ as long as the bandwidth is large enough. For the following, the states in the reservoirs are equally spaced between frequencies $\omega_{\min}$ and $\omega_{\max}$ and then shifted by the asymmetry parameter δ, given $k \in \{1, \ldots, N_r\}$: $\omega_k = (\omega_{\min} - \Delta/2) + \Delta k + \delta/2$ where the state spacing is $\Delta = (\omega_{\max} - \omega_{\min})/N_r$ and the couplings are constant $v_k = \sqrt{8J^2/(2\pi N_r)}$. Taking the limit as $N_r \to \infty$, the integral of the single particle Green's functions yields for $\mathcal{L}$

$$\Sigma^{r(a)}(\omega) = \frac{8J^2}{2\pi W} \ln\left(\frac{\omega - \delta/2 - \omega_{\min} \mp \imath\gamma/2}{\omega - \delta/2 - \omega_{\max} \mp \imath\gamma/2}\right), \quad \text{(C6)}$$

and similarly for $\mathcal{R}$. Here the bandwidth is $W = \omega_{\max} - \omega_{\min}$. The calculation of the steady-state current then continues as previously.

At zero temperature ($\beta \to \infty$), expanding the current for large bandwidth (and small V, δ), the leading terms are

$$I \approx \frac{eV(8J^2/W)}{\hbar\pi[\gamma + 2(8J^2/W)]} - \frac{8e\delta\gamma J^2}{\pi W^3}. \quad \text{(C7)}$$

The first term gives the linear response current. For small γ, it is $eV/2\pi\hbar$. The second term gives a residual, unphysical current. So long as the asymmetry is small compared to the bandwidth or γ is sufficiently small, this term will be negligible compared to the linear response contribution. Moreover, to accurately calculate results in the Landauer regime for steady states, γ needs to be of the order of W/N_r (this condition depends on the details of the setup, as discussed in the main text regarding the small γ regime). Thus, so long as

$$\gamma \approx W/N_r \ll W^3 V/\delta J^2 \hbar \quad \text{(C8)}$$

then the simulation will accurately predict steady state behavior. In other words, one needs $N_r \gg J^2\delta\hbar/W^2V$ (or, for our particular example in the main text, $N_r \gg \delta/V$), which is a condition that is basically always fulfilled, to ensure the simulation gives accurate results in the Landauer regime. This covers both small and intermediate regimes discussed throughout the text.

Appendix D: Linear Reservoir with Equally Spaced States

The process of discretization of the states can have a large effect on the calculated current when using the master equation, particularly when the bias window lies completely in a gap between states. As an example, in the 1D lattice case, the extended reservoir portion of H can be directly diagonalized via a sine transformation. Thus, if one wished to study a large—but still finite—extended reservoir driving a current through a time-dependent junction, one could increase N_r by increasing the size of the reservoir in real space, a fact that also applies in higher dimensions. However, it can be more useful to instead take N_r states evenly spaced in energy and increase N_r by decreasing the spacing. That is, given $k \in \{1, \ldots, N_r\}$, $\omega_k = -(W/2 + \Delta/2) + \Delta k$, where the state spacing is $\Delta = W/N_r$, places the states evenly within the energy band (note that $W = 4J$ for this model). In order for this to represent the 1D extended reservoir, the coupling constants to the system need to incorporate the local density of states for the real-space lattice at the boundary with the system. We can write the couplings with a single index, v_k for $k \in \mathcal{L}, \mathcal{R}$ (instead of v_{ki}), as the coupling to the system's boundaries only depend on k. Using this notation, the couplings

$$v_k = 2(4J^2 - \omega_k^2)^{1/4}\sqrt{J/2\pi N_r}, \quad \text{(D1)}$$

have a factor of $\sqrt{4J^2 - \omega_k^2}$, which is the local density of states for the 1D lattice. In the $N_r \to \infty$ limit, this choice of state discretization recovers the semi-infinite, real-space lattice.

Appendix E: Single-Site Reservoir Rate Equation

When the broadening γ is much smaller than the state spacing $\approx W/N_r$ and for a non-interacting system, conservation of energy requires that each electron entering the system from a site with a given energy ϵ_k also leave the system from the a site with the same energy. This allows the current to be broken into contributions from pairs of extended reservoir states, which can be calculated from a system of rate equations. We shall examine a three site system which consists of a single site from each of $\mathcal{L}$ and $\mathcal{R}$ (indexed by k) and a single site in $\mathcal{S}$. Further, we will assume different relaxation rates on the left and right, $\gamma_{\mathcal{L}}$ and $\gamma_{\mathcal{R}}$ respectively to make the appearance of a "reduced γ" clear.

When γ is small, the effect of the environment $\mathcal{E}_{\mathcal{L}(\mathcal{R})}$ is to relax each extended state to a target filling, denoted by $f^{\mathcal{L}(\mathcal{R})}$, at a rate $\gamma_{\mathcal{L}(\mathcal{R})}$. This current due to relaxation is proportional to the difference between the onsite occupation of the reservoir state, $n^{\mathcal{L}(\mathcal{R})}$, and $f^{\mathcal{L}(\mathcal{R})}$. In linear response, the current from $\mathcal{E}_{\mathcal{L}}$ and the current into $\mathcal{E}_{\mathcal{R}}$ are

$$I_k^{\mathcal{L}} \approx e\gamma_{\mathcal{L}}(f_k^{\mathcal{L}} - n_k^{\mathcal{L}}), \qquad I_k^{\mathcal{R}} \approx e\gamma_{\mathcal{R}}(n_k^{\mathcal{R}} - f_k^{\mathcal{R}}). \quad \text{(E1)}$$

The current between $\mathcal{S}$ and the reservoir states is proportional to a rate parameter σ times the difference in occupations:

$$I_k^{\mathcal{LS}} \approx e\sigma(n_k^{\mathcal{L}} - n^{\mathcal{S}}), \qquad I_k^{\mathcal{SR}} \approx e\sigma(n^{\mathcal{S}} - n_k^{\mathcal{R}}). \quad \text{(E2)}$$

The value of σ is related to the coupling between the reservoir and system and describes the particle flow rate. In the general case, this would be a function of the system-reservoir coupling and the total Green's function. For the example system, however, σ scales as $1/N_r$. In linear response, the conductance is independent of J and W, so the sum of the total current from all N_r states must be a constant.

In the steady state, all four of these currents—$I_k^{\mathcal{L}}$, $I_k^{\mathcal{R}}$, $I_k^{\mathcal{LS}}$, $I_k^{\mathcal{SR}}$—must be equal, giving the solution

$$I_k = \frac{e\sigma}{\left(2 + \sigma\frac{\gamma_{\mathcal{L}}+\gamma_{\mathcal{R}}}{\gamma_{\mathcal{L}}\gamma_{\mathcal{R}}}\right)}(f_k^{\mathcal{L}} - f_k^{\mathcal{R}}). \quad \text{(E3)}$$

The quantity $\gamma_{\mathcal{L}}\gamma_{\mathcal{R}}/(\gamma_{\mathcal{L}}+\gamma_{\mathcal{R}})$ is the "reduced γ" between the two reservoirs and is simply $\gamma/2$ when the relaxation rates are equal. The small γ ($\gamma \ll \sigma$) approximation for Eq. (E3) with equal relaxation rates is $I_k \approx e(\gamma/2)(f_k^{\mathcal{L}} - f_k^{\mathcal{R}})$, as used in the main text.

This rate argument can be extended to the full $N_r \neq 1$ system by solving a system of $4N_r$ equations for each of the incoming and outgoing currents and then equating the total current into $n_{\mathcal{S}}$, $\sum_k I_k^{\mathcal{LS}}$, with the total current leaving $\sum_k I_k^{\mathcal{SR}}$. When $\gamma_{\mathcal{L}} = \gamma_{\mathcal{R}} = \gamma$, this yields the current through the system as

$$I \approx e\gamma/2 \sum_k (f_k^{\mathcal{L}} - f_k^{\mathcal{R}})\frac{\sigma}{\sigma+\gamma}. \quad \text{(E4)}$$

For small γ, this recovers the expression in the main text. Additionally, the occupation of the central site, $n^{\mathcal{S}}$, is found to be equal to the mean target filling, $\sum_k (f_k^{\mathcal{L}} + f_k^{\mathcal{R}})/(2N_r)$. With a symmetrical distribution of reservoir state energies and bias window (as in the case of the main text), then the central site onsite density is 1/2.

Lastly, a full solution involving a different σ_k for each reservoir mode is obtainable. Assuming that the σ_k are symmetric between the left and right sides, the total current flow recovers Eq. (E4) to the lowest order of γ. The relaxation strength limits the total current through the system and, so long as γ is sufficiently small, then the current is independent of the system-reservoir coupling.

* mpz@nist.gov

[1] H. Haug and A.-P. Jauho, *Quantum kinetics in transport and optics of semiconductors* (Springer, 1996).

[2] A. Jauho, N. Wingreen, and Y. Meir, Phys. Rev. B **50**, 5528 (1994).

[3] Y. Meir and N. S. Wingreen, Phys. Rev. Lett. **68**, 2512 (1992).

[4] This can also be seen working with directly with a diagonalized linear reservoir when $v_k = J\mathcal{U}_{k1}$, where $\mathcal{U}$ is the unitary transformation that diagonalizes the single particle Hamiltonian.
